# MedSAM/MedSAM2 Feature Fusion: Enhancing nnUNet for 2D TOF-MRA Brain Vessel Segmentation

**DOI:** 10.3390/jimaging11060202

**Published:** 2025-06-18

**Authors:** Han Zhong, Jiatian Zhang, Lingxiao Zhao

**Affiliations:** 1 School of Biomedical Engineering, Division of Life Sciences and Medicine, University of Science and Technology of China, Hefei 230026, China; zh2687400933@mail.ustc.edu.cn (H.Z.); zjt20174025@mail.ustc.edu.cn (J.Z.); 2 Suzhou Institute of Biomedical Engineering and Technology, Chinese Academy of Sciences, Suzhou 215613, China

**Keywords:** TOF-MRA, brain vessel segmentation, nnUNet, MedSAM, MedSAM2

## Abstract

Accurate segmentation of brain vessels is critical for diagnosing cerebral stroke, yet existing AI-based methods struggle with challenges such as small vessel segmentation and class imbalance. To address this, our study proposes a novel 2D segmentation method based on the nnUNet framework, enhanced with MedSAM/MedSAM2 features, for arterial vessel segmentation in time-of-flight magnetic resonance angiography (TOF-MRA) brain slices. The approach first constructs a baseline segmentation network using nnUNet, then incorporates MedSAM/MedSAM2’s feature extraction module to enhance feature representation. Additionally, focal loss is introduced to address class imbalance. Experimental results on the CAS2023 dataset demonstrate that the MedSAM2-enhanced model achieves a 0.72% relative improvement in Dice coefficient and reduces HD95 (mm) and ASD (mm) from 48.20 mm to 46.30 mm and from 5.33 mm to 4.97 mm, respectively, compared to the baseline nnUNet, showing significant enhancements in boundary localization and segmentation accuracy. This approach addresses the critical challenge of small vessel segmentation in TOF-MRA, with the potential to improve cerebrovascular disease diagnosis in clinical practice.

## 1. Introduction

Stroke, the third leading cause of death globally after ischemic heart disease and COVID-19 [1], is characterized by high rates of disability and mortality. It usually manifests itself as arterial stenosis, atherosclerosis, abnormal branching patterns, and altered circular anastomotic function [2,3]. These pathological changes reduce blood supply capacity and increase ischemic risk, making a precise assessment of the cerebrovascular structure crucial for clinical diagnosis and treatment. Unlike contrast-enhanced MRA (CE-MRA), TOF-MRA is a non-invasive, contrast-free alternative that avoids risks linked to gadolinium-based agents [4]. Importantly, TOF-MRA shows diagnostic performance comparable to CE-MRA for detecting non-severe cerebrovascular stenosis and intracranial aneurysms [5,6], making it the preferred screening modality for low-risk populations. Given the growing concerns about the security and privacy of medical data (particularly clinical imaging and segmentation tasks) [7], however, traditional manual segmentation methods are limited by low efficiency and accuracy, with strong subjectivity and poor reproducibility, making them inadequate for meeting clinical demands in rapid and precise quantitative analyses such as lumen stenosis rate calculation [8]. The inherent physical limitations of non-contrast MRA result in reduced spatial resolution, lower signal-to-noise ratio, and increased artifacts [9,10]. These limitations pose significant challenges for AI-based TOF-MRA segmentation tasks. Although deep learning technologies have made substantial progress in medical image segmentation, particularly in automation and precision [11], research on TOF-MRA-based cerebral artery segmentation still faces challenges due to scarce annotated datasets [12] and consequent limitations in algorithm generalizability and robustness. Therefore, research on AI-based segmentation methods for TOF-MRA images is particularly crucial. Improving the accuracy and efficiency of TOF-MRA image segmentation can better address evolving clinical requirements, providing stronger support for the diagnosis and treatment of cerebrovascular diseases.

In recent explorations to overcome the limitations of traditional segmentation methods, the medical image segmentation field has witnessed the emergence of two innovative approaches with complementary characteristics: domain-specific architectures represented by nnUNet [13] and general segmentation models exemplified by MedSAM/MedSAM2 [14,15]. The nnUNet framework demonstrates remarkable stability in specific medical imaging tasks through its adaptive network configuration and hyperparameter optimization mechanisms, automatically optimizing its architecture for diverse datasets and tasks. This eliminates manual tuning while achieving higher precision than conventional UNet architectures, solidifying its status as a state-of-the-art (SOTA) method in medical image segmentation [13]. In contrast, vision transformer-based MedSAM series models harness their large-scale pretraining to develop powerful feature representation capabilities. This enables consistently robust performance across challenging clinical scenarios, including TOF-MRA cerebrovascular segmentation tasks characterized by structurally complex vascular networks and significant noise interference. The effective integration of nnUNet and MedSAM/MedSAM2 creates a synergistic framework that combines nnUNet’s superior local feature extraction capacity with MedSAM2’s global modeling advantages. Using high-quality feature representations from MedSAM/MedSAM2, the proposed nnUNet-MedSAM/MedSAM2 framework achieves efficient and precise medical image segmentation, demonstrating significant performance improvements over individual approaches. The main contributions are as follows:

1. We propose a hybrid nnUNet-MedSAM/MedSAM2 architecture that integrates nnUNet with MedSAM/MedSAM2 to synergize their complementary strengths: MedSAM/MedSAM2 provides generalized feature representation through large-scale pretraining, while nnUNet offers task-specific adaptation. The hybrid design enhances multi-scale feature extraction and improves robustness against imaging noise and artifacts [14,15].
2. We propose an enhanced nnUNet training strategy by incorporating Focal Loss to address the extreme class imbalance (e.g., <5% vascular pixels in TOF-MRA volumes). This effectively mitigates the severe imbalance between cerebrovascular structures and background tissues in TOF-MRA neuroimaging, enabling more balanced feature representation learning for both dominant background regions and sparse vascular targets.
3. We introduce a FrequencyLoRA module before feature fusion between nnUNet and MedSAM/MedSAM2, which captures global structures through spectral enhancement while achieving efficient local feature optimization via low-rank bottleneck compression, balancing noise robustness with computational efficiency. After feature fusion, we employ an AttentionGate module that synergistically combines channel attention (global context modeling) with spatial attention (local structure perception) to enhance feature selection and noise suppression simultaneously.

## 2. Methods

### 2.1. Architecture Overview

**Figure 1:**
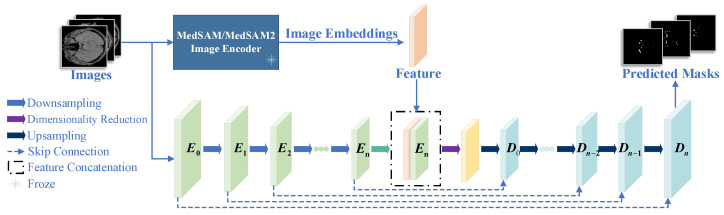
Framework overview. MedSAM/MedSAM2 embeddings fuse with the nnUNet encoder‘s specific stages, then progress through the decoder ($D_{0}$–$D_{n}$) with skip connections for mask prediction. The green arrow indicates the impending initiation of feature fusion.

This paper proposes the nnUNet-MedSAM/MedSAM2 framework, which effectively integrates the powerful feature extraction capabilities of MedSAM/MedSAM2 with the automated advantages of nnUNet. In this study, we employ fine-tuned MedSAM/MedSAM2 encoders to extract features from TOF-MRA images, which effectively capture both global contextual information and local detailed features in medical images, thereby providing high-quality feature inputs for nnUNet. Figure 1 illustrates the overall framework of the cerebral artery segmentation network. The input image is processed through two parallel image encoders, MedSAM/MedSAM2 and nnUNet, to obtain respective feature embeddings. Subsequently, the feature fusion occurs at distinct hierarchical levels: MedSAM interacts exclusively with the final encoder stage of nnUNet, whereas MedSAM2 establishes multi-scale connections spanning both intermediate and terminal encoding stages.

### 2.2. Feature Extraction Network in MedSAM/MedSAM2

**Figure 2:**
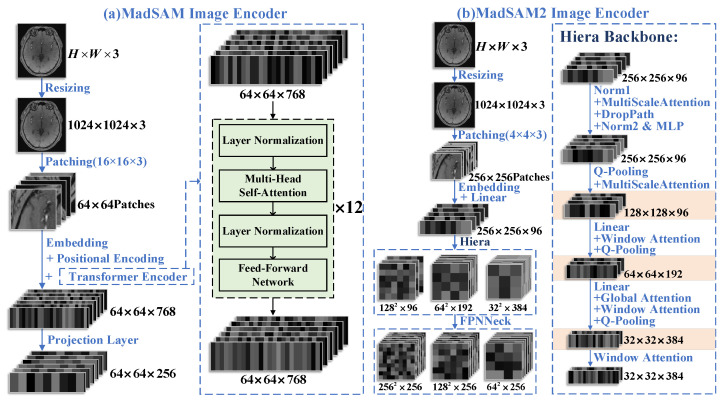
Encoder architectures comparison: (**a**) MedSAM image encoder includes patch processing, embedding, multi-layer transformer blocks (with LayerNorm, multi-head self-attention, and MLP), and feature output. (**b**) MedSAM2 image encoder uses a Hiera backbone with hierarchical attention (e.g., Q-Pooling and window attention) for multi-scale feature extraction.

As shown in Figure 2a, the MedSAM image encoder adopts a vision transformer (ViT) architecture [16], which consists of three main components: patch embedding, transformer encoding, and a feature projection layer (to reduce the output dimension), collectively transforming input images into high-dimensional feature embeddings. First, the input image is resized to a fixed dimension of 1024×1024×3 and divided into 64×64 non-overlapping patches, each with a size of 16×16×3. Each patch is then linearly projected (embedding) into a 1D vector of length 768, while a learnable 1D positional encoding (position embedding) is incorporated to preserve spatial information. These position-aware feature vectors are subsequently processed by the transformer encoder to generate a high-dimensional feature embedding with dimensions of 64×64×768. Finally, the feature projection layer reduces the feature dimension from 768 to 256 through 1×1 convolutions, producing the final feature map with dimensions of 64×64×256.

The transformer encoder in MedSAM employs a classical stack architecture, with its processing pipeline illustrated on the right side of Figure 2a. The input feature map (64×64×768) first undergoes layer normalization (LayerNorm) to stabilize the data distribution, followed by processing through the core multi-head self-attention (MHSA) module. This module performs 12 consecutive attention computation iterations to capture global spatial dependencies. After each attention computation, another layer normalization is applied before nonlinear feature transformation via a feed-forward network (FFN). Throughout this process, the spatial resolution (64×64) and channel dimension (768) of the feature map remain unchanged. The final output features contain rich, deep representations with comprehensive global contextual information.

As illustrated in Figure 2b, MedSAM2’s image encoder represents a significant improvement over MedSAM’s architecture. Unlike MedSAM’s simple convolutional and normalization-based neck module, MedSAM2 employs an FPN-based FPNNeck [17] that effectively integrates multi-level features. Compared to MedSAM’s single-scale feature embedding, MedSAM2 generates three distinct scale feature maps: 128×128×96, 64×64×192, and 32×32×384. This multi-scale representation enables more effective capture of both fine local details and comprehensive multi-scale characteristics in medical images.

As shown in the right side of Figure 2b, the Hiera encoder [18] in MedSAM2 employs a hierarchical transformer architecture for efficient feature extraction through multi-stage downsampling and hybrid attention mechanisms. The four-stage structure begins with layer normalization and a multi-scale attention module maintaining 256×256×96 resolution, enhanced by DropPath regularization and MLP blocks, followed by stride-2 query pooling (Q-Pooling) and another multi-scale attention module to generate 128×128×96 features. In stage 2, linear projection expands channels to 192, where Window Attention reinforces edge-texture features before Q-Pooling reduces resolution to 64×64×192. Stage 3 further projects channels to 384, alternately applying global attention and window attention for joint local–global modeling, with subsequent Q-Pooling producing 32×32×384 features. The final stage retains this dimensionality while streamlining computation through window attention-only blocks. Collectively, Hiera achieves hierarchical feature abstraction via progressive Q-Pooling and channel doubling (96→192→384), constructing a multi-scale pyramid that progressively integrates high-resolution local details with low-resolution global semantics.

### 2.3. nnUNet Segmentation Framework

**Figure 3:**
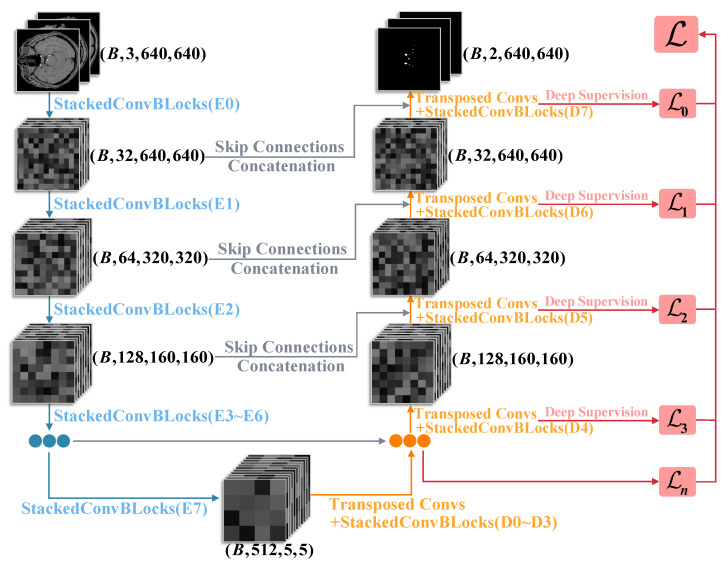
Architecture overview of nnUNet. The model processes 640×640 input images through stacked convolutional blocks ($E_{0}$–$E_{7}$) for hierarchical feature extraction, with skip connections and transposed convolutions enabling precise segmentation through U-Net encoder–decoder structure. Deep supervision ($L_{0}$–$L_{n}$) is applied at multiple levels.

As illustrated in Figure 3, the nnUNet architecture adopts an end-to-end encoder–decoder structure for medical image segmentation. The encoder path (left) performs four-level downsampling through stacked convolutional blocks (StackedConvBLOCKs $E_{0}$–$E_{7}$), progressively compressing the input image (B, 3, 640, 640) into high-level feature representations (B, 512, 5, 5). The decoder path (right) employs transposed convolutions (Transposed Convs) for stepwise upsampling, integrating multi-scale feature fusion via skip connections and concatenation with corresponding encoder-level feature maps. Additionally, the network incorporates deep supervision [19], where intermediate predictions ($D_{0}$–$D_{7}$) are generated at each decoding stage and optimized through a multi-level loss function to enhance segmentation performance.

**Figure 4:**
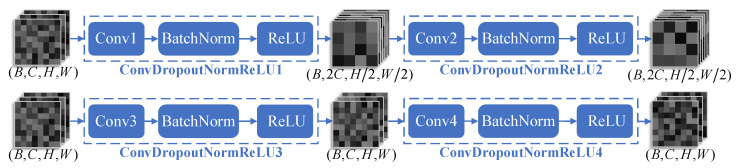
Schematic of nnUNet’s stacked convolutional blocks in encoder–decoder architecture. (**Top**) Encoder’s downsampling StackedConvBlocks ($E_{n}$). (**Bottom**) Decoder’s StackedConvBlocks ($D_{n}$). Blue dashed frames demarcate modular units, with arrows indicating feature flow directions.

Figure 4 illustrates the structure of the StackedConvBLOCK, where each StackedConvBLOCK consists of two ConvDropoutNormReLU modules. Conv1, Conv2, Conv3, and Conv4 all use 3 × 3 convolutional kernels. During the image encoding process, the Conv1 layer in the ConvDropoutNormReLU1 module employs a stride of 2 and doubles the number of output channels relative to the input, simultaneously achieving both channel dimension expansion and spatial resolution halving. Meanwhile, the Conv2 layer in the ConvDropoutNormReLU2 module uses a stride of 1 while maintaining identical input and output channel dimensions, thus preserving both channel count and spatial dimensions. During the image decoding process, Conv3 and Conv4 in ConvDropoutNormReLU3 and ConvDropoutNormReLU4 both have a stride of 1, performing feature transformations without changing the number of feature channels or spatial dimensions. This allows the module to work with subsequent Transposed Convs [20] to halve the number of feature channels and double the spatial dimensions.

### 2.4. Feature Extraction Network in MedSAM/MedSAM2

**Figure 5:**
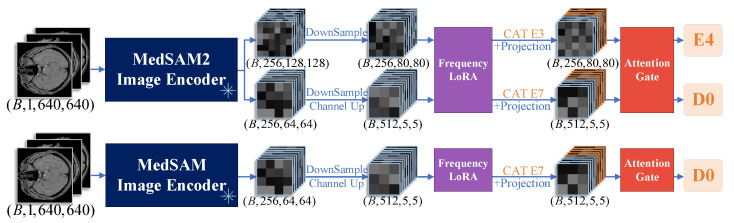
Comparison of two feature fusion methods for MedSAM2 and nnUNet. (**Top**) MedSAM2 features fused at both $E_{3}$ and $E_{7}$ encoder stages of nnUNet. (**Bottom**) MedSAM features fused only at $E_{7}$ stage. Both pipelines process input images (640×640) through independent encoding paths with downsampling, channel adjustment (Channel Up), and feature enhancement (FrequencyLoRA), followed by CAT and projection operations.

As shown in Figure 5, the feature fusion between nnUNet and MedSAM2 proceeds as follows: the image first passes through the MedSAM2 encoder to obtain multi-scale feature embeddings. To avoid introducing excessive high-frequency details and noise while reducing computational load, this paper does not employ early feature fusion based on the (B, 256, 256, 256) features from MedSAM2. Therefore, two specific embeddings with shapes (B, 256, 128, 128) and (B, 256, 64, 64) undergo downsampling and downsampling with channel doubling operations, respectively. This process transforms them into (B, 256, 80, 80) and (B, 512, 5, 5) features, aligning their dimensional scales with those of stage $E_{3}$ and $E_{7}$ in the nnUNet encoder. These processed features then pass through FrequencyLoRA modules for enhanced representation before being concatenated with corresponding nnUNet features via channel-wise concatenation (CAT) modules. Subsequent 1×1 convolutional projection reduces dimensionality, followed by adaptive feature selection through AttentionGate modules, with the final fused features output to the next processing stage. In the feature fusion method between nnUNet and MedSAM, besides the single-scale features from MedSAM being fused only with the seventh stage ($E_{7}$) of the nnUNet image encoder, all other processes are completely consistent with the feature fusion method of nnUNet and MedSAM2.

**Figure 6:**
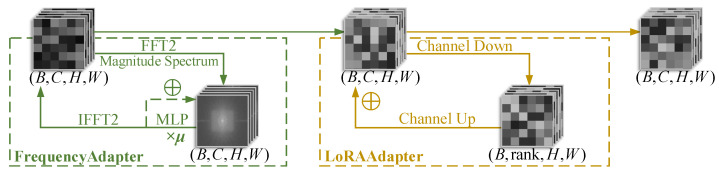
Schematic of the FrequencyLoRA module. (**Left**) FrequencyAdapter processes input (B,C,H,W) through FFT2 spectrum analysis and MLP-based feature enhancement (green dashed frame). (**Right**) LoRAAdapter performs low-rank adaptation via channel down/up operations (yellow dashed frame). Both modules employ residual connections (dotted arrows) to maintain original features (see Section 2.4 for implementation details).

Figure 6 illustrates the architecture of the FrequencyLoRA module, which integrates both frequency-domain enhancement and low-rank adaptation(LoRA) [21] through two key submodules: FrequencyAdapter and LoRAAdapter. The module first uses the fast Fourier transform (FFT) [22] to extract frequency-domain information from feature maps, where amplitude spectrum analysis captures global high-frequency details. These spectral features are modulated by a lightweight MLP network, then incorporated into the original frequency-domain features through a residual connection (parameterized by μ) to enhance the high-frequency components, before being transformed back to the spatial domain via the inverse Fourier transform. Simultaneously, the low-rank adaptation mechanism (parameterized by rank) is introduced, implementing parameter-efficient local feature refinement via a bottleneck structure with dimension reduction–expansion operations. This dual-path design effectively preserves the spatial inductive bias of convolutional neural networks while augmenting the model’s representational capacity for fine-grained image details and structural information through frequency-domain priors. The synergistic integration of these mechanisms achieves enriched feature representation while maintaining computational efficiency.

**Figure 7:**
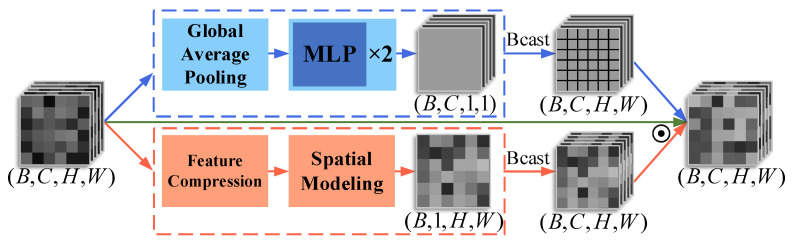
Architecture of the AttentionGate module. The input tensor (B,C,H,W) undergoes parallel processing: (1) channel attention (blue path) via global average pooling and two-layer MLP and (2) spatial attention (orange path) through feature compression and spatial modeling. Both branches output broadcast-compatible weights that are fused through element-wise multiplication, preserving the original tensor dimensions. Dashed frames distinguish processing stages while arrows indicate data flow.

Figure 7 demonstrates that the AttentionGate module achieves intelligent feature recalibration through parallel channel and spatial attention mechanisms, contrasting with CBAM’s [23] sequential approach while preserving its dual-attention philosophy. The module first establishes inter-channel dependencies via global average pooling followed by two consecutive MLP layers, where LayerNorm enhances training stability to generate channel weights that emphasize important feature channels. Concurrently, a multi-scale convolutional structure extracts spatial context information to produce a spatial weight map highlighting key regions. These dual attention weights are then fused through element-wise multiplication. This synergistic dual-attention design enables the module to adaptively suppress irrelevant background noise while enhancing local features, thereby significantly improving the model’s capability for semantic information extraction from images.

The formula for element-wise multiplication to fuse the dual attention weights is as follows:

(1) $$O_{i,j,k,l}=I_{i,j,k,l}\cdot \sigma \left({\hat{W}}_{c_{i,j,k,l}}\right)\cdot \sigma \left({\hat{W}}_{s_{i,j,k,l}}\right),$$

where $I_{i,j,k,l}$ and $O_{i,j,k,l}$ denote the input and output feature tensors, respectively. ${\hat{W}}_{c_{i,j,k,l}}$ and ${\hat{W}}_{s_{i,j,k,l}}$ represent the channel and spatial attention weights, normalized to [0, 1] through the sigmoid function to achieve a soft attention mechanism.

**Figure 8:**
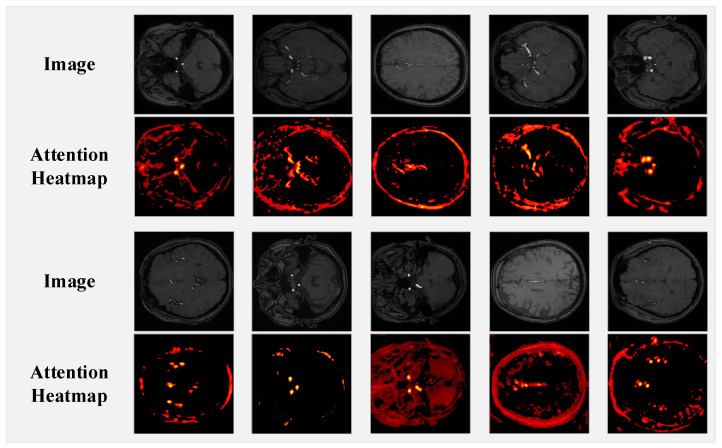
TOF-MRA slices and their spatial attention heatmaps in the AttentionGate module.

Figure 8 displays ten sets of TOF-MRA brain slices and their corresponding spatial attention heatmaps from the AttentionGate module in nnUNet-MedSAM2. The specific steps are as follows: first, we extract the attention weights from the AttentionGate module and convert them into NumPy arrays. Then, these raw attention maps are upsampled to the original image dimensions (640×640) using bilinear interpolation to preserve spatial relationships. Finally, for enhanced visualization, the values are normalized to [0, 1] and mapped to a red-to-orange gradient heatmap, with an empirical threshold of 0.5 to suppress background regions (values below the threshold are set to black), thereby improving the clarity of vascular structures. The heatmaps are presented in a gradient from red to orange, where higher brightness indicates greater model attention to that region, while the black background represents areas with no significant attention. Our observations reveal that the model’s high-attention regions primarily focus on cross-sections of cerebral arteries in the TOF-MRA slices. This suggests that the spatial attention mechanism enhances the model’s focus on key anatomical structures of the cerebral vasculature. By weighting feature responses in vascular regions, it improves the model’s ability to identify and distinguish cerebrovascular lesions.

### 2.5. Focal Loss-Based Optimization for nnUNet

Due to the spatial morphological characteristics of vascular cross-sections in 2D images, even after the cropping step (retaining only the non-zero pixel regions of interest, ROIs) in the nnUNet preprocessing pipeline, the segmentation task still suffers from class imbalance caused by the low pixel proportion and scattered distribution of target structures. This leads to the model’s inability to explicitly distinguish between easy-to-classify samples (e.g., large-diameter cerebral trunk arteries such as the M1 segment of the middle cerebral artery) and hard-to-classify samples (e.g., peripheral small vessels like perforating arteries). To address this issue, our study introduces the focal loss [24], weighted and combined with the original cross-entropy loss, to amplify gradient contributions from hard samples and enhance the model’s sensitivity to fine vessels. The focal loss formula is as follows:

(2) $$L_{\mathrm{Focal}}=-\frac{1}{N}\sum_{i=1}^{N}\alpha_{t}{\left(1-p_{t}\right)}^{\gamma }\log\left(p_{t}\right),$$

where $p_{t}$ represents the predicted probability of the *i*-th pixel belonging to its ground-truth class. For positive samples, $p_{t}=p_{i}$, while for negative samples, $p_{t}=1-p_{i}$, where $p_{i}$ denotes the predicted probability of the model for the target class. The term $\alpha_{t}$ is a class-weighting parameter: for positive samples, $\alpha_{t}=\alpha$, and for negative samples, $\alpha_{t}=1-\alpha$. The modulating factor γ adjusts the relative weighting between easy and hard samples. Finally, the focal loss for the entire image is computed as the average of the focal loss values across all pixels.

As shown in Equation (3), a novel cross-entropy loss function is formed by weighted combination of $L_{CE}$ and $L_{Focal}$ through parameters α and β, which is further combined with Dice loss to construct the $L_{Enhanced}$:

(3) $$L_{Enhanced}=\lambda_{1}\cdot \left(\alpha L_{CE}+\beta L_{Focal}\right)+\lambda_{2}\cdot L_{Dice}.$$

Dice loss focuses on enhancing model segmentation performance for small targets and regions with complex shapes. By measuring the overlap between predicted segmentation and ground-truth segmentation, Dice loss enables the model to make more precise predictions along boundaries and contours. In Equation (4), Dice loss is the complement of the Dice coefficient, where $y_{i}$ represents the true label of pixel *i* (0 for background, 1 for target class), $p_{i}$ is the model’s predicted probability that pixel *i* belongs to the target class, and *N* is the total number of pixels in the image.

(4) $$L_{Dice}=1-\frac{2\cdot \sum_{i=1}^{N}y_{i}\cdot p_{i}}{\sum_{i=1}^{N}y_{i}+\sum_{i=1}^{N}p_{i}}.$$

Cross-entropy loss is a widely used loss function for multi-class classification problems. It evaluates model performance by measuring the discrepancy between true labels and predicted probabilities. Equation (5) shows the conventional cross-entropy loss function, where $p_{i,y_{i}}$ denotes the model’s predicted probability for the true class $y_{i}$ at pixel *i*. The image-level loss is computed as the average over all pixels, with *N* being the total number of pixels.

(5) $$L_{CE}=-\frac{1}{N}\sum_{i=1}^{N}\log\left(p_{i,y_{i}}\right).$$

### 2.6. Dataset and Preprocessing

This study used the publicly available CAS2023 dataset [25], comprising TOF-MRA 3D imaging data (in NIIGZ format) from 100 stroke patients. During preprocessing, all 3D images were cropped along the Z-axis and converted into 2D slices, ultimately generating 14,189 2D images (in PNG format), with strict adherence to ensuring that the training and test sets originated from distinct 3D imaging samples.

In the five-fold cross-validation of the nnUNet training framework, each iteration reserved one fold (16 cases) as the validation set while the remaining folds (64 cases) served as the training set for model training, yielding the corresponding fold-specific weights. The final segmentation result was obtained by averaging the inference outputs from the five trained models. For the five-fold cross-validation in nnUNet, this study refined the framework’s original 2D dataset partitioning strategy: first, the training set (80 cases) was divided into five groups at the 3D image level, with each group containing complete 3D case data before 2D slice generation. This stratified partitioning approach effectively prevented slices from the same case from appearing in both training and validation sets, eliminating data leakage risks and ensuring scientific rigor.

**Figure 9:**
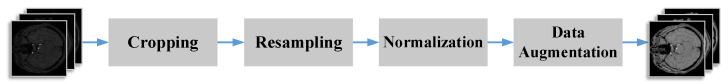
Preprocessing pipeline for MRA images in nnUNet.

In this study, the built-in automated preprocessing pipeline of the nnUNet framework, extensively validated across diverse medical imaging datasets, was used in this study. As illustrated in Figure 9, the MRA image preprocessing method initially crops input images to retain only non-zero regions, focusing on ROIs and reducing computational load. Subsequently, the images were resampled on the basis of the dataset’s median pixel spacing to ensure precise spatial context capture using third-order spline interpolation for images and nearest-neighbor interpolation for masks. Each 2D image then underwent independent Z-score normalization. Finally, data augmentation techniques, including random rotation, scaling, elastic deformation, and mirror flipping, were applied.

### 2.7. Computational Environment and Parameters

The specifications and parameters of each component in the experimental platform are listed in Table 1. Additionally, to control for extraneous variables, all experiments mentioned in this paper were conducted with the GPU random seed set to 3407. Regarding the methodology proposed in this study, the maximum number of training epochs for the nnUNet model was uniformly set at 250 in all experiments, and the model was trained using SGD with an initial learning rate, momentum (0.99), weight decay, and Nesterov acceleration, while the learning rate was adjusted by a polynomial scheduler (PolyLR) with an exponent of 0.9 over the total training epochs. Furthermore, prior to the frozen image encoder of MedSAM/MedSAM2 being engaged in feature fusion, the model undergoes fine-tuning to optimize its feature extraction performance. Specifically, MedSAM/MedSAM2 is pre-trained under default configurations using the identical training dataset as nnUNet, with both models trained for 50 epochs.

Regarding the hyperparameters in the proposed module, to avoid affecting the information in the original features, the channel reduction factor in the FrequencyLoRA module is set to 8, μ is set to 0.2, and the rank is set to 4. Additionally, in the improved loss function $L_{\mathrm{Enhanced}}$, both α and β are set to 0.5, while $\lambda_{1}$ and $\lambda_{2}$ are both set to 1. For $L_{\mathrm{Focal}}$, the class weight (α) and the weight for adjusting hard/easy samples (γ) are set to [0.25, 0.75] and 2, respectively.

## 3. Results

### 3.1. Evaluation Metrics

For a comprehensive evaluation of the experimental results, this article uses four key metrics: Dice (%), IoU (%), Hausdorff distance 95% (HD95, mm), and average symmetric surface distance (ASD, mm) to assess the performance of the model. Within the binary classification framework of this study, Dice (%) and IoU (%) are formally defined as follows:

(6) $$\mathrm{Dice}\left(\%\right)=\frac{2|P\cap G|}{\left|P|+|G\right|}\times 100\%,$$

(7) $$\mathrm{IoU}\left(\%\right)=\frac{\left|P\cap G\right|}{\left|P\cup G\right|}\times 100\%,$$

where *P* denotes the predicted point set, and *G* represents the ground-truth point set. |P| and |G| represent the total pixel counts of the predicted and ground-truth labels, respectively. |P∩G| denotes the number of pixels that intersect between the predicted and ground-truth labels, while |P∪G| indicates their union pixel count. Dice (%) quantifies the overlap between the predicted and ground-truth labels by calculating the ratio of twice the number of overlapping pixels to the total number of pixels in both regions. It incorporates the concept of harmonic mean, making it more sensitive to small target segmentation. The definitions of HD95 (mm) and ASD (mm) are as follows:

(8) $$HD95\;\left(\mathrm{mm}\right)=\mathrm{Percentile}\left(\max\left\{\underset{p\in \partial P}{\sup}\underset{g\in \partial G}{\inf}d\left(p,g\right),\underset{g\in \partial G}{\sup}\underset{p\in \partial P}{\inf}d\left(g,p\right)\right\},95\%\right),$$

(9) $$ASD\;\left(\mathrm{mm}\right)=\frac{1}{\left|\partial P|+|\partial G\right|}\left(\sum_{p\in \partial P}\min_{g\in \partial G}d\left(p,g\right)+\sum_{g\in \partial G}\min_{p\in \partial P}d\left(g,p\right)\right),$$

where ∂P and ∂G respectively represent the surface contour point sets of the predicted segmentation result and the ground-truth annotation, |∂P| and |∂G| are the total numbers of pixel points in the predicted contour and real contour, respectively, d(p,g) is the Euclidean distance between point *p* and point *g*, and mind(p,g) represents the minimum distance from point *p* to the real contour. 95% denotes the 95th percentile of the distance distribution to mitigate the influence of extreme outliers. HD95 (mm), calculated as the 95th percentile of the maximum bidirectional Hausdorff distance between the predicted and ground-truth contours, quantifies the maximum boundary error in segmentation results. This metric emphasizes localized under-segmented regions or protruding structures. In contrast, the ASD (mm) measures the mean nearest-neighbor distance between all corresponding points on the predicted and true contours, reflecting the global average boundary alignment accuracy. Unlike HD95, ASD focuses on the general deviation of the boundary rather than on localized extreme discrepancies.

In addition, to comprehensively evaluate the performance of the 2D vascular segmentation model in critical clinical scenarios, this study also employs three core metrics for comparative analysis at the pixel level: precision, recall, and F1-score. Among these, recall is used to assess the model’s risk of missing small vessels, thereby avoiding the oversight of critical lesions due to false negatives. Precision addresses the issue of over-segmentation that may lead to false positives, preventing misjudgments in hemodynamics or vascular structures. The F1-score, as the harmonic mean of the two, provides a balanced measure to mitigate the limitations of relying on a single metric. The precision, recall, and F1-score are defined as follows:

(10) $$\mathrm{Precision}=\frac{TP}{TP+FP},\;\mathrm{Recall}=\frac{TP}{TP+FN},\;F1=\frac{2\cdot \mathrm{Precision}\cdot \mathrm{Recall}}{\mathrm{Precision}+\mathrm{Recall}},$$

where TP represents the number of pixels in a single image that are predicted as positive and are actually positive, FP represents the number of pixels predicted as positive but are actually negative, and FN represents the number of pixels predicted as negative but are actually positive. Precision is the ratio of the number of samples predicted as positive that are actually positive to the total number of samples predicted as positive. Recall is the ratio of the number of samples predicted as positive that are actually positive to the total number of samples that are actually positive. The F1-score is the harmonic mean of precision and recall.

All performance metrics (Dice, IoU, etc.) were calculated by first determining image-specific values and then computing macro-averages, thereby preserving balanced contributions from all individual images in the dataset.

### 3.2. Comparison with Baseline and Competing Methods

To validate the effectiveness of the proposed method, this paper evaluates the performance of mainstream approaches, including UNet [26], TransUNet [27], SwinUNet [28], and nnUNet, on the CAS2023 dataset, and conducts a comparative assessment with the proposed method across four metrics: Dice (%), IoU (%), HD95 (mm), and ASD (mm). To reasonably reflect the generalizability and stability of the model in the overall data set, the comparative experiments adopted a five-fold cross-validation, with results recorded in the form of mean ± standard deviation. As shown in Table 2, the proposed methods (nnUNet-MedSAM and nnUNet-MedSAM2) outperform the baseline model nnUNet across all four metrics and demonstrate significant advantages over UNet, TransUNet, and SwinUNet. Compared to the nnUNet baseline, the proposed nnUNet-MedSAM achieves relative improvements of 0.63% and 0.93% in Dice and IoU, respectively, while reducing HD95 and ASD from 48.20mm to 46.51mm and 5.33mm to 5.03mm, respectively. The proposed nnUNet-MedSAM2 further enhances performance, showing relative improvements of 0.72% in Dice and 1.00% in IoU while reducing HD95 from 48.20 mm to 46.30 mm and ASD from 5.33 mm to 4.97 mm. In particular, the proposed methods show particularly significant improvements in HD95 (mm) and ASD (mm), indicating substantial advances in boundary localization and segmentation accuracy. It should be mentioned that among the two proposed feature fusion strategies, nnUNet-MedSAM2 slightly outperforms nnUNet-MedSAM. This suggests that the multi-scale feature fusion approach based on the Hiera image encoder in MedSAM2 is superior to the single-scale feature fusion method employed in MedSAM.

**Figure 10:**
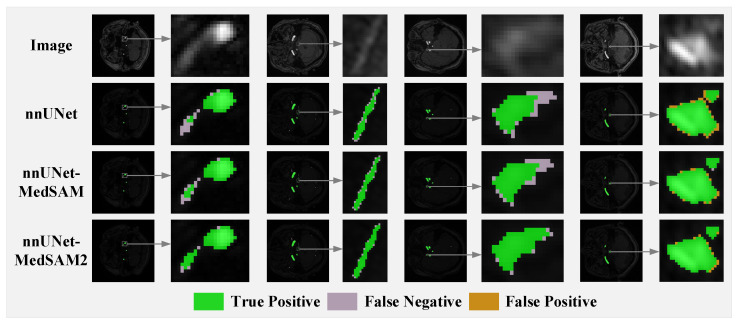
The segmentation performance of nnUNet and nnUNet-MedSAM/MedSAM2: correctly segmented pixels are shown in green, false negative pixels are in light purple, and false positive pixels are in yellow.

Due to the significant advantages of nnUNet and the proposed methods over UNet, SwinUNet, and TransUNet in this task, this paper focuses on comparing the details of segmentation between nnUNet and the proposed methods. As shown in Figure 10, compared to the nnUNet baseline model, the proposed nnUNet-MedSAM/MedSAM2 achieves higher segmentation accuracy for small blood vessels and more precise boundary localization. Additionally, thanks to multi-scale feature fusion, the segmentation accuracy of nnUNet-MedSAM2 is higher than that of nnUNet-MedSAM.

From Table 3, it can be observed that the proposed methods (nnUNet-MedSAM and nnUNet-MedSAM2) outperform UNet, SwinUNet, TransUNet, and the baseline model nnUNet across all three metrics: precision, recall, and F1-score. Notably, the pixel-level F1-score is numerically equivalent to the Dice coefficient. The analysis reveals that the two proposed methods achieve more significant improvements in recall (mean recall increases from 83.51% for nnUNet to 83.99% for nnUNet-MedSAM and 84.37% for nnUNet-MedSAM2), while precision remains relatively stable (mean precision for nnUNet, nnUNet-MedSAM, and nnUNet-MedSAM2 is 87.76%, 88.06%, and 87.83%, respectively). This indicates that the optimization strategy effectively reduces the missed detection rate of fine vessels without introducing significant false segmentation. Such an improvement better aligns with clinical needs, as it avoids the risk of missing critical lesion information due to undetected vessels.

Further analysis shows that nnUNet-MedSAM achieves a slightly higher mean precision (88.06%) compared to nnUNet-MedSAM2 (87.83%). This discrepancy may stem from the fact that while the multi-stage frequency-domain enhancement in nnUNet-MedSAM2 improves recall, the redundancy of high-frequency features in shallow layers could slightly increase false positives. In contrast, the single fusion stage in MS helps maintain feature consistency more effectively.

### 3.3. Ablation Study on Loss Functions

To validate the effectiveness of the introduction of Focal Loss, ablation experiments were conducted on both proposed feature fusion methods by removing Focal Loss. As shown in Table 4, the removal of focal loss led to a decline in the metrics of both feature fusion methods. Specifically, for the nnUNet-MedSAM method, Dice and IoU relatively decreased by 0.18% and 0.27%, respectively, while HD95 and ASD increased from 46.51 mm to 47.26 mm and from 5.03 mm to 5.31 mm, respectively. Similarly, for the nnUNet-MedSAM2 method, Dice and IoU relatively decreased by 0.24% and 0.29%, respectively, while HD95 and ASD increased from 46.30 mm to 47.04 mm and from 4.97 mm to 5.26 mm, respectively. These results demonstrate that the incorporation of focal loss effectively enhances the model’s performance in the task of brain vessel segmentation based on 2D TOF-MRA images.

The marginal improvement brought by focal loss (as shown in Table 4, where removing Focal Loss only reduced the average Dice coefficient of nnUNet-MedSAM2 by 0.20) suggests two hypotheses: (1) Dice loss has already effectively mitigated the class imbalance issue [29], leading to diminishing returns; (2) the current hyperparameters (α and γ) may not be fully optimized for this dataset, failing to fully adapt to its characteristics. Hyperparameter optimization could enable the model to more flexibly accommodate complex imbalanced scenarios and unleash greater potential [30].

### 3.4. Ablation Study on Feature Fusion

To validate the effectiveness of introducing MedSAM/MedSAM2 feature fusion, ablation experiments were conducted on the proposed feature fusion methods. As shown in Table 5, removing the MedSAM/MedSAM2 feature fusion modules led to a decline in all four evaluation metrics. Specifically, for the nnUNet-MedSAM method, Dice and IoU decreased by 0.28% and 0.38%, respectively, while HD95 and ASD increased from 46.51 mm to 47.42 mm and 5.03 mm to 5.29 mm, respectively. Similarly, for the nnUNet-MedSAM2 method, Dice and IoU decreased by 0.37% and 0.44%, respectively, while HD95 and ASD increased from 46.30 mm to 47.42 mm and 4.97 mm to 5.29 mm, respectively. These results demonstrate that the MedSAM/MedSAM2-based feature fusion approach effectively enhances feature representation and improves model performance.

## 4. Discussion

This study achieved precise segmentation of cerebral arterial vessels in 2D TOF-MRA brain image slices. By designing and introducing a feature fusion module for MedSAM/MedSAM2 that incorporates frequency-domain feature enhancement and dual channel–spatial attention mechanisms, and by combining the focal loss with the original cross-entropy loss and Dice loss in a weighted manner, the segmentation accuracy of the baseline model nnUNet was effectively improved, particularly in significantly reducing spatial error metrics such as HD95 (mm) and ASD (mm) (e.g., the nnUNet-MedSAM2 method reduced HD95 and ASD from 48.20 mm to 46.30 mm and from 5.33 mm to 4.97 mm, respectively). These results validate the advantage of the proposed method in capturing subtle boundary differences in vascular structures. Additionally, from a clinical perspective, the proposed method significantly improves the recall rate despite only a marginal increase in segmentation accuracy (mean recall increases from 83.51% for nnUNet to 83.99% for nnUNet-MedSAM and 84.37% for nnUNet-MedSAM2), effectively reducing the false negative rate and lowering the risk of missed detection in clinical practice.

Further analysis demonstrates that the performance improvement contributed by MedSAM/MedSAM2 feature fusion significantly outweighs that of the focal loss-based training strategy optimization. Specifically, in the nnUNet-MedSAM2 approach, the multi-stage and multi-scale feature fusion strategy enhances the model’s feature representation capability. Although nnUNet is the SOTA method, it has nearly saturated performance on conventional evaluation metrics (e.g., Dice coefficient) when sufficient data are available. Our experiments show that integrating large-model features such as MedSAM/MedSAM2 can further enhance the segmentation performance of nnUNet.

In summary, while the absolute performance improvement of the proposed method is modest, this fusion strategy provides a novel approach to overcoming the limitations of traditional single-model architectures. Integrating the general anatomical prior knowledge from large models with the task-specific focus of dedicated segmentation models enables the extraction of deeper feature correlations that remain underutilized in image data. Additionally, ablation studies (Section 3.3) confirm that designing tailored training strategies (e.g., focal loss) for vessel segmentation is also a critical factor in performance enhancement.

It should be noted that the effectiveness of feature fusion heavily relies on the quality of features generated by large models, which typically require extensive fine-tuning to fully adapt to the target task. In addition, the incorporation of large models significantly increases computational costs and reduces inference speed. Moreover, the design methodology proposed in this paper can also be extended to 3D segmentation tasks. Therefore, future work should focus on the following: (1) Design an automated fine-tuning framework for MedSAM/MedSAM2, incorporating an adaptive feature fusion mechanism to optimize cross-modal feature representation through dynamic weight adjustment, thereby enhancing the model’s adaptation efficiency in target data domains; (2) To address the high inference latency of nnUNet-MedSAM/MedSAM2 (a more than 100% increase compared to the baseline), propose a hybrid optimization solution: combine model compression techniques (e.g., quantization-aware training (QAT), structured pruning) with hardware acceleration (TensorRT deployment) and optimize computational graph parallelism to significantly reduce inference latency and GPU memory usage while maintaining segmentation accuracy; (3) Leveraging MedSAM2’s 3D segmentation capability, design a dual-dimensional feature fusion framework: integrate MedSAM2’s 3D spatial priors with nnUNet for multi-scale feature fusion in 3D segmentation tasks, while combining the 2D segmentation branch of nnUNet-MedSAM2 proposed in this work to fully exploit the ensemble advantages of nnUNet’s 2D and 3D capabilities, further enhancing boundary sensitivity and topological consistency in nnUNet’s 3D segmentation.

## Figures and Tables

**Table 1:** Hardware configuration of the experimental platform.

Component	Specification
Operating System	Ubuntu 22.04.4 LTS
CPU	13th Gen Intel® Core™ i9-13900KF
GPU	NVIDIA® GeForce RTX 4090
RAM	64 GB DDR5 4800 MT/s

**Table 2:** Performance comparison of different models on the CAS2023 dataset in terms of Dice (%), IoU (%), HD95 (mm), and ASD (mm). Ours (MS) denotes our method of fusing features between nnUNet and MedSAM, while Ours (MS2) represents the feature fusion of nnUNet with MedSAM2.

Method	Dice (%)	IoU (%)	HD95 (mm)	ASD (mm)
UNet	81.92 ± 1.18	70.84 ± 1.70	51.13 ± 11.21	5.49 ± 1.03
SwinUNet	82.09 ± 1.00	71.04 ± 1.49	50.63 ± 11.82	5.46 ± 0.81
TransUNet	82.21 ± 1.08	71.19 ± 1.57	50.37 ± 12.13	5.40 ± 0.92
nnUNet	83.89 ± 0.92	73.86 ± 1.39	48.20 ± 10.89	5.33 ± 0.76
Ours (MS)	84.42 ± 0.97	74.55 ± 1.46	46.51 ± 12.02	5.03 ± 0.77
Ours (MS2)	84.49 ± 0.99	74.60 ± 1.49	46.30 ± 11.67	4.97 ± 0.80

**Table 3:** Performance comparison of different models on the CAS2023 dataset in terms of precision (%), recall (%), and F1-score (%). Ours (MS) denotes our method of fusing features between nnUNet and MedSAM, while Ours (MS2) represents the feature fusion of nnUNet with MedSAM2.

Method	Precision (%)	Recall (%)	F1-Score (%)
UNet	86.12 ± 1.55	81.52 ± 1.33	81.92 ± 1.18
SwinUNet	86.20 ± 1.37	81.61 ± 1.12	82.09 ± 1.00
TransUNet	86.22 ± 1.39	81.69 ± 1.21	82.21 ± 1.08
nnUNet	87.76 ± 1.31	83.51 ± 1.06	83.89 ± 0.92
Ours (MS)	88.06 ± 1.37	83.99 ± 1.08	84.42 ± 0.97
Ours (MS2)	87.83 ± 1.35	84.37 ± 1.13	84.49 ± 0.99

**Table 4:** Ablation study comparing the full method and its variant without focal loss (FL) using four segmentation metrics: Dice (%), IoU (%), HD95 (mm), and ASD (mm).

Method	Dice (%)	IoU (%)	HD95 (mm)	ASD (mm)
Full (MS) ^1^	84.42 ± 0.97	74.55 ± 1.46	46.51 ± 12.02	5.03 ± 0.77
w/o FL (MS) ^2^	84.27 ± 0.94	74.35 ± 1.40	47.26 ± 11.39	5.31 ± 0.76
Full (MS2) ^3^	84.49 ± 0.99	74.60 ± 1.49	46.30 ± 11.67	4.97 ± 0.80
w/o FL (MS2) ^4^	84.29 ± 0.93	74.38 ± 1.41	47.04 ± 11.62	5.26 ± 0.75

^1^ The full method (nnUNet-MedSAM) with Focal Loss (FL). ^2^ The full method (nnUNet-MedSAM) with Focal Loss (FL) removed. ^3^ The full method (nnUNet-MedSAM2) with Focal Loss (FL). ^4^ The full method (nnUNet-MedSAM2) with Focal Loss (FL) removed.

**Table 5:** Ablation study comparing the complete method (nnUNet-MedSAM/MedSAM2) with its feature-fusion-removed variants (the FrequencyLoRA module before fusion and the AttentionGate module after fusion are also removed together) evaluated on four segmentation metrics: Dice (%), IoU (%), HD95 (mm), and ASD (mm).

Method	Dice (%)	IoU (%)	HD95 (mm)	ASD (mm)
Full (MS) ^1^	84.42 ± 0.97	74.55 ± 1.46	46.51 ± 12.02	5.03 ± 0.77
Full (MS2) ^2^	84.49 ± 0.99	74.60 ± 1.49	46.30 ± 11.67	4.97 ± 0.80
w/o FF ^3^	84.18 ± 0.97	74.27 ± 1.47	47.42 ± 11.46	5.29 ± 0.76

^1^ The full method (nnUNet-MedSAM) with integrated MedSAM feature fusion. ^2^ The full method (nnUNet-MedSAM2) with integrated MedSAM2 feature fusion. ^3^ The full method (nnUNet-MedSAM/MedSAM2) with feature fusion module removed.

## Data Availability

The data presented in this study are available in zenodo at https://zenodo.org/records/7839970 (accessed on 22 May 2025). These data were derived from the following resources available in the public domain: https://codalab.lisn.upsaclay.fr/competitions/9804 (accessed on 22 May 2025).
